# Guapiaçu virus, a new insect-specific flavivirus isolated from two species of *Aedes* mosquitoes from Brazil

**DOI:** 10.1038/s41598-021-83879-6

**Published:** 2021-02-25

**Authors:** Geovani de Oliveira Ribeiro, Antonio Charlys da Costa, Danielle Elise Gill, Edcelha Soares D’Athaide Ribeiro, Marlisson Octavio da S. Rego, Fred Julio Costa Monteiro, Fabiola Villanova, Juliana Silva Nogueira, Adriana Yurika Maeda, Renato Pereira de Souza, Roozbeh Tahmasebi, Vanessa S. Morais, Ramendra Pati Pandey, V. Samuel Raj, Sirle Abdo Salloum Scandar, Fernanda Gisele da Silva Vasami, Leandro Guaraglia D’Agostino, Paulo César Maiorka, Xutao Deng, Maurício Lacerda Nogueira, Ester Cerdeira Sabino, Eric Delwart, Élcio Leal, Mariana Sequetin Cunha

**Affiliations:** 1 Institute of Biological Sciences, Federal University of Pará, Belém, Pará 66075-000 Brazil; 2 Institute of Tropical Medicine, University of São Paulo, São Paulo, SP 05403-000 Brazil; 3 Public Health Laboratory of Amapa-LACEN/AP, Health Surveillance Superintendence of Amapa, Rua Tancredo Neves, 1.118, Macapá, AP CEP 68905-230 Brazil; 4 Vector-Borne Diseases Laboratory, Virology Center, Adolfo Lutz Institute, São Paulo, SP 01246-000 Brazil; 5 Centre for Drug Design Discovery and Development (C4D), SRM University, Delhi-NCR, Rajiv Gandhi Education City, Sonepat, Haryana India; 6 Superintendence for Control of Endemic Diseases (SUCEN), São José do Rio Preto, SP Brazil; 7 Department of Pathology, Faculty of Veterinary Medicine, University of São Paulo, São Paulo, SP Brazil; 8 Vitalant Research Institute, 270 Masonic Avenue, San Francisco, CA 94118-4417 USA; 9 Department Laboratory Medicine, University of California San Francisco, San Francisco, CA 94143 USA; 10 Faculdade de Medicina de São José do Rio Preto (FAMERP), São José do Rio Preto, SP Brazil

**Keywords:** Evolutionary genetics, Genomics, Virology

## Abstract

Classical insect-flaviviruses (cISFVs) and dual host-related insect-specific flavivirus (dISFV) are within the major group of insect-specific flavivirus. Remarkably dISFV are evolutionarily related to some of the pathogenic flavivirus, such as Zika and dengue viruses. The Evolutionary relatedness of dISFV to flavivirus allowed us to investigate the evolutionary principle of host adaptation. Additionally, dISFV can be used for the development of flavivirus vaccines and to explore underlying principles of mammalian pathogenicity. Here we describe the genetic characterization of a novel putative dISFV, termed Guapiaçu virus (GUAPV). Distinct strains of GUAPV were isolated from pools of *Aedes terrens* and *Aedes scapularis* mosquitoes. Additionally, we also detected viral GUAPV RNA in a plasma sample of an individual febrile from the Amazon region (North of Brazil). Although GUAPV did not replicate in tested mammalian cells, 3′UTR secondary structures duplication and codon usage index were similar to pathogenic flavivirus.

## Introduction

Members of the *Flavivirus* genus belongs to the *Flaviviridae* family, and possess a single-stranded, positive-sense RNA genome of approximately 11 kb^1^. Phylogenetic analysis has demonstrated that flaviviruses cluster according to their host preference range: insect-specific flaviviruses (ISFs), dual-host tick-borne flaviviruses (TBFVs), mosquito-borne flaviviruses (MBFV), and viruses with no known vector (NKV)^2,3^.

MBFV is the largest group that includes some of the most important human pathogens, such as dengue virus (DENV), yellow fever virus (YFV), and Zika virus (ZIKV). MBFV is transmitted to vertebrates through biting of hematophagous arthropods and are considered therefore to be dual-host flaviviruses^4^.

An increasing number of flaviviruses that infect only insects (i.e., insect-specific flaviviruses—ISFVs) have been identified during the last two decades^5–7^. ISFVs infect hematophagous *Diptera* and replicate in mosquito cells, but are unable to replicate in mammalian cells or infect vertebrates^8^. Based on their phylogenetic and antigenic relationships, ISFVs can be separated into two distinct groups. The classical insect-specific flaviviruses (cISFs), such as cell fusing agent (CFAV), Culex flavivirus (CxFV), and Kamiti River (KRV) viruses are the largest group, and they are phylogenetically distinct from all other known flaviviruses^9^.

The cISFV is a more phylogenetically divergent flavivirus group that may represent an ancient flavivirus lineage. The evolutionary relationship between arthropod-specific viruses and arboviruses still is unclear. An evolution from arthropod-specific viruses has been assumed for the genus *Flavivirus*, as several phylogenetic studies have shown that many pathogenic viruses probably evolved from being insect-specific virus to dual host viruses^10–12^. However, other recent studies have recently discovered a novel group of ISFVs, named dual host-affiliated insect-specific flaviviruses (dISFs)^13,14^. The dISFs are phylogenetically and antigenically mostly related to the flavivirus vertebrate pathogens within the MBVV group^6^, thus suggesting that vertebrate tropism is convergent, acquired at least two times in flaviviruses^15,16^. Therefore, further studies are needed to understand the evolutionary origin of pathogenic flavivirus and ISFV as well as the restriction of flavivirus replication in mammalian cells. In the current manuscript we report the detection, isolation, nearly complete genome sequencing, and phylogenetic assignment of a novel dISFV named Guapiaçu virus (GUAPV). GUAPV was isolated in pools of *Aedes *spp., captured in Guapiaçu municipality in northern São Paulo state (southeast Brazil). We also detected GUAPV in a human plasma sample from Macapá city (Amapá state in north Brazil) derived from an individual suffering from fever and fatigue.

## Results

### Virus isolation and viral growth

Two pools, one from *Aedes terrens* with a total of six mosquitoes and another with two specimens of *Aedes scapularis*, were collected in Guapiaçu city on March 15th, 2017. Both pools tested positive for flavivirus using polyclonal antibodies and negative using DENV and YFV monoclonal antibodies. RT-qPCR was negative for ZIKV and WNV, and positive for *Flavivirus* genus. In parallel, a positive RT-qPCR for flavivirus genus followed by a small NS5 gene analysis resulted in the identification of unknown flaviviruses in the sample obtained from a patient residing in Macapá city, Amapá state, north Brazil. We further characterized these unknown flaviviruses using next-generation sequencing.

**Figure 1:**
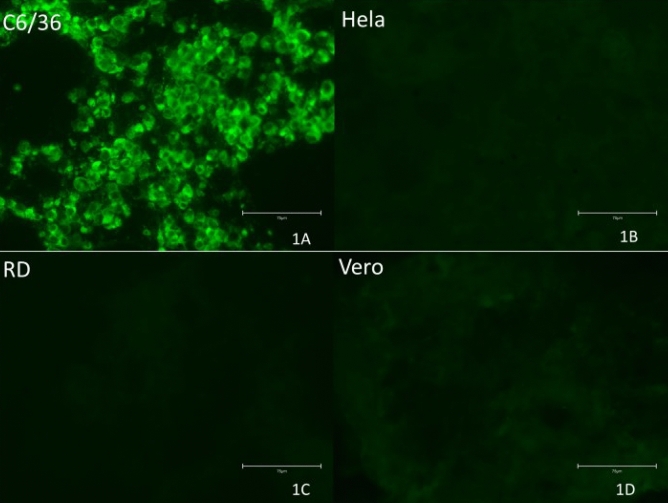
Guapiaçu virus IFA results on day 7 after inoculation. 1A: C6/36 infected cells; 1B: Hela cells; 1C: RD cells; 1D: Vero cells. Scale in 75μM (40x).

During the experiment of viral growth in different cell lines, no cytopathic effect was detected in mammalian cells, neither in C6/36 cells. The pan-flavivirus RT-qPCRs performed showed that there was a Ct decrease only in the mosquito derived cell C6/36, indicating an increase of one viral log, as previously demonstrated for this assay^17^, and also consistent with most flavivirus growth in vitro. All mammalian cells showed an increase in Ct values (See Supplementary files S1 and S2). IFA performed in the 7th day was positive in C6/36, and negative in mammalian cells, showing that viral replication has occurred only in mosquito-derived cells (Fig. 1).

### Genomic analysis

**Figure 2:**
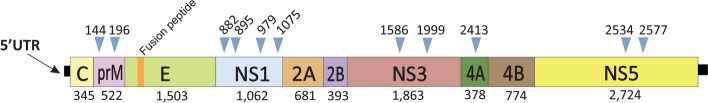
Genomic organization of GUAPV. The number under the box indicates the nucleotide length of each peptide and the blue triangle indicates the position of potential N-glycosylation sites.

**Figure 3:**
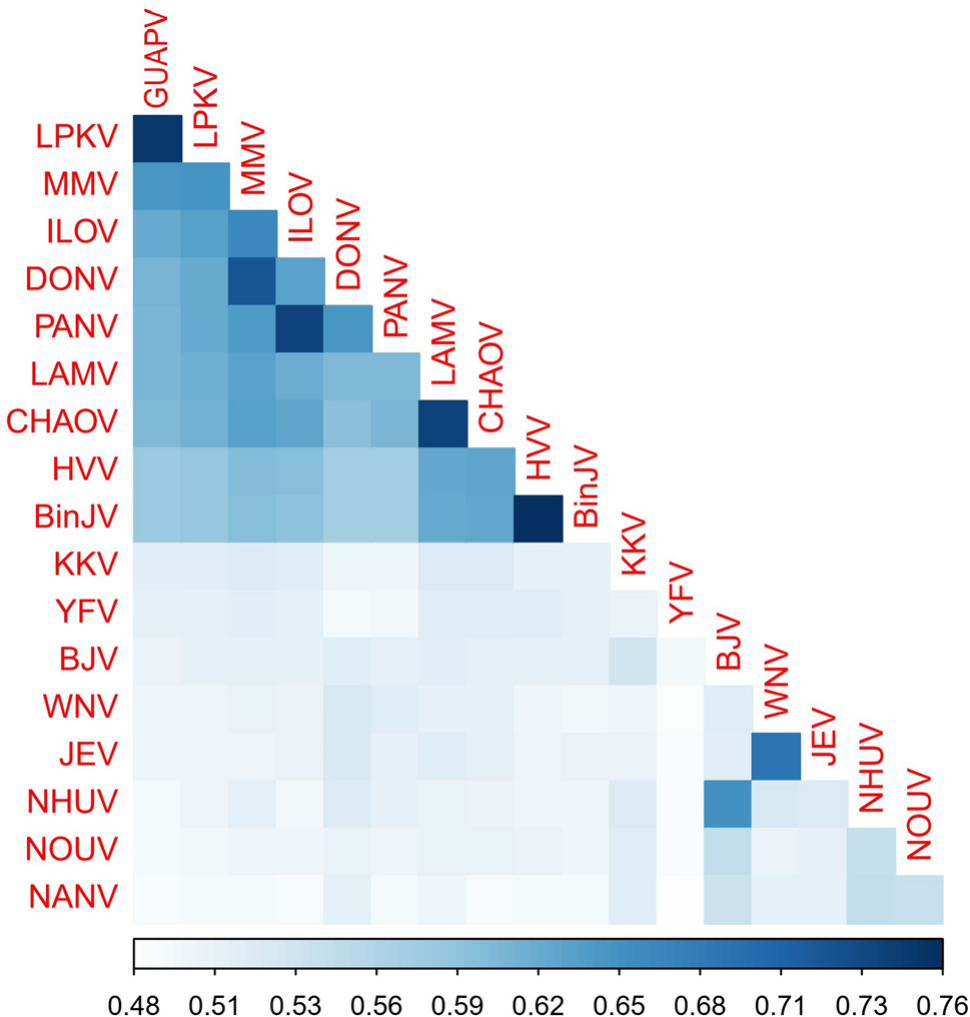
Nucleotide pairwise identity of distinct flaviviruses. Values are indicated by color shading. Pairwise identity matrix was generated from complete genomes nucleotide sequences using p-distance model and 1000 bootstrap replication in MEGAX software version 10.0.5. GUAPV (Guapiaçu virus), LPKV (Long Pine Key virus), MMV (Marisma mosquito virus), ILOV (Ilomantsi virus), DONV (Donggang virus), PANV (Panmunjeom flavivirus), LAMV, CHAOV (Chaoyang virus), HVV (Hidden valley virus), BinJV (Binjari virus), KKV (Kampung Karu virus), YFV (Yellow fever virus), BJV (Barkedji virus), WNV (West nile virus), JEV (Japanese encephalitis virus), NHUV (Nhumirim virus), NOUV (Nienokoue virus) and NANV (Nanay virus).

The near complete sequences of GUAPV were determined from deep sequencing. Coding sequence analysis revealed a single open reading frame of 10,314 nucleotides in length, encoding a polyprotein of 3438 amino. The sequences were deposited in GenBank under the accession numbers MK908097–MK908103 (Fig. 2). Nucleotide comparison was performed between GUAPV and dISFV, cISFV, and MFBV members (Fig. 3). The nucleotide sequence of GUAPV was found to be divergent from any of the previously known flaviviruses, sharing from 36 to 75% of identity with other flaviviruses in NS5 (the most conserved protein). When amino acid was analyzed, GUAPV harbors 95% of similarity in NS5 protein with Long pine key virus (LPKV), whereas, with other flavivirus the similarity ranged from 41 to 76%.

### Phylogenetic analysis

**Figure 4:**
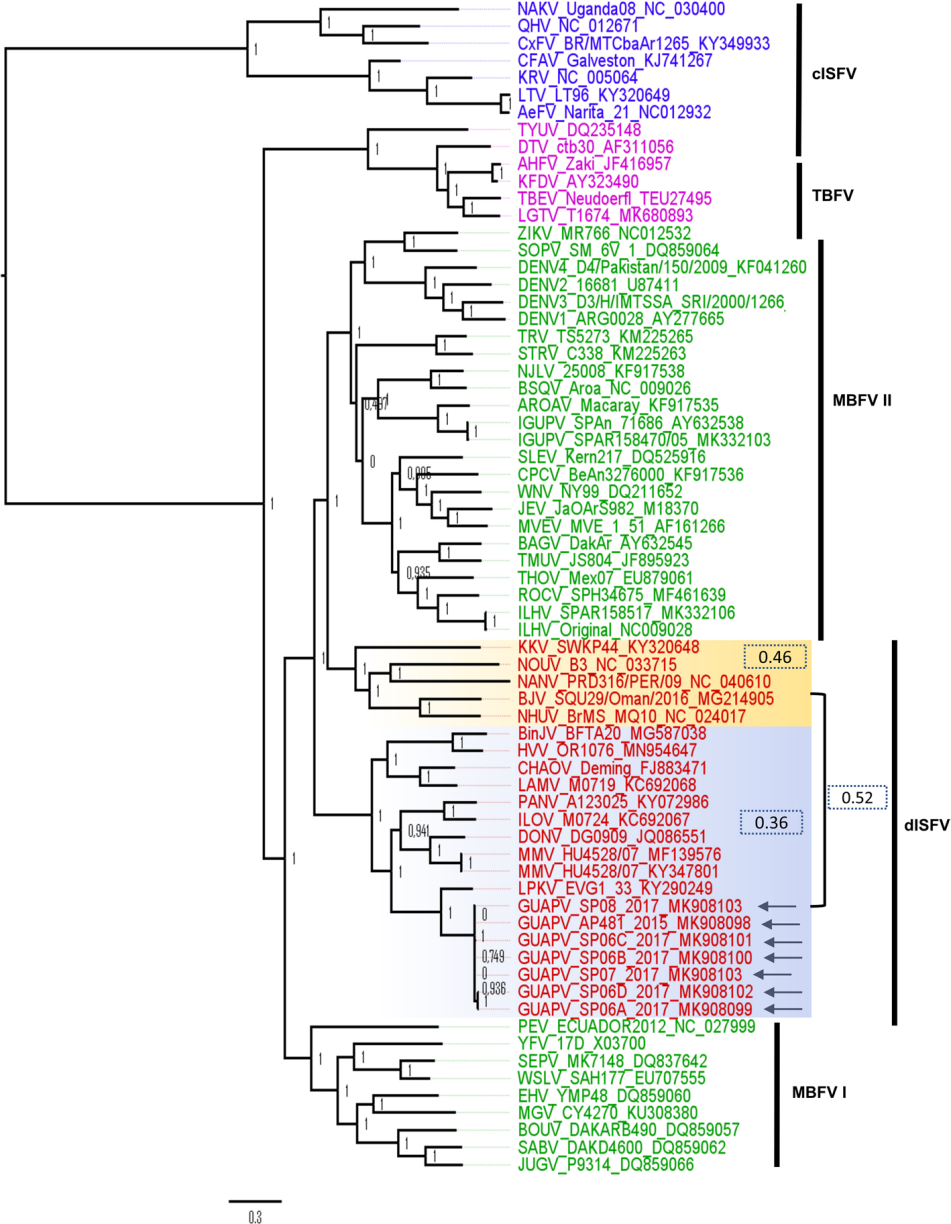
Maximum likelihood phylogeny of the genus Flavivirus members. Strain names and GenBank accession numbers are given after the names of the viruses. The GUAPV sequences of this study are represented by arrows. The genetic distance based on p-distance was computed within and between dISFs clades. Highlighted in blue are classical insect-specific flaviviruses (cISFs), in pink are tick-borne flavivirus (TBFV), in green are mosquito-borne flavivirus (MBFV) and red are dual host-related insect-specific flavivirus (dISFV). The tree is rooted at the midpoint and approximate Likelihood-Ratio values are indicated in the nodes of the tree.

The topology of the phylogenetic tree based on the ORFs of available sequences of other flaviviruses agrees with works published previously^9,18,19^ and demonstrates the segregation of the major clusters consisting of the mosquito-borne, tick-borne, and insect-specific flaviviruses (Fig. 4). As expected, the cISFs, represented by CxFV, CFAV, Aedes Flavivirus (AeFV), and others, clustered in a clade basal to all other member species of the *Flavivirus* genus. GUAPV group within the dual host-related insect-specific flavivirus (dISFV) clade and has a close relationship with LPKV (100% statistical support). Interestingly, two paraphyletic clades with 52% genetic distance between them were observed in topology, suggesting that dISFV emerged at two independent events, as it was previously proposed^15,20^.

### Codon Adaptation Indexes of viral coding genes

**Figure 5:**
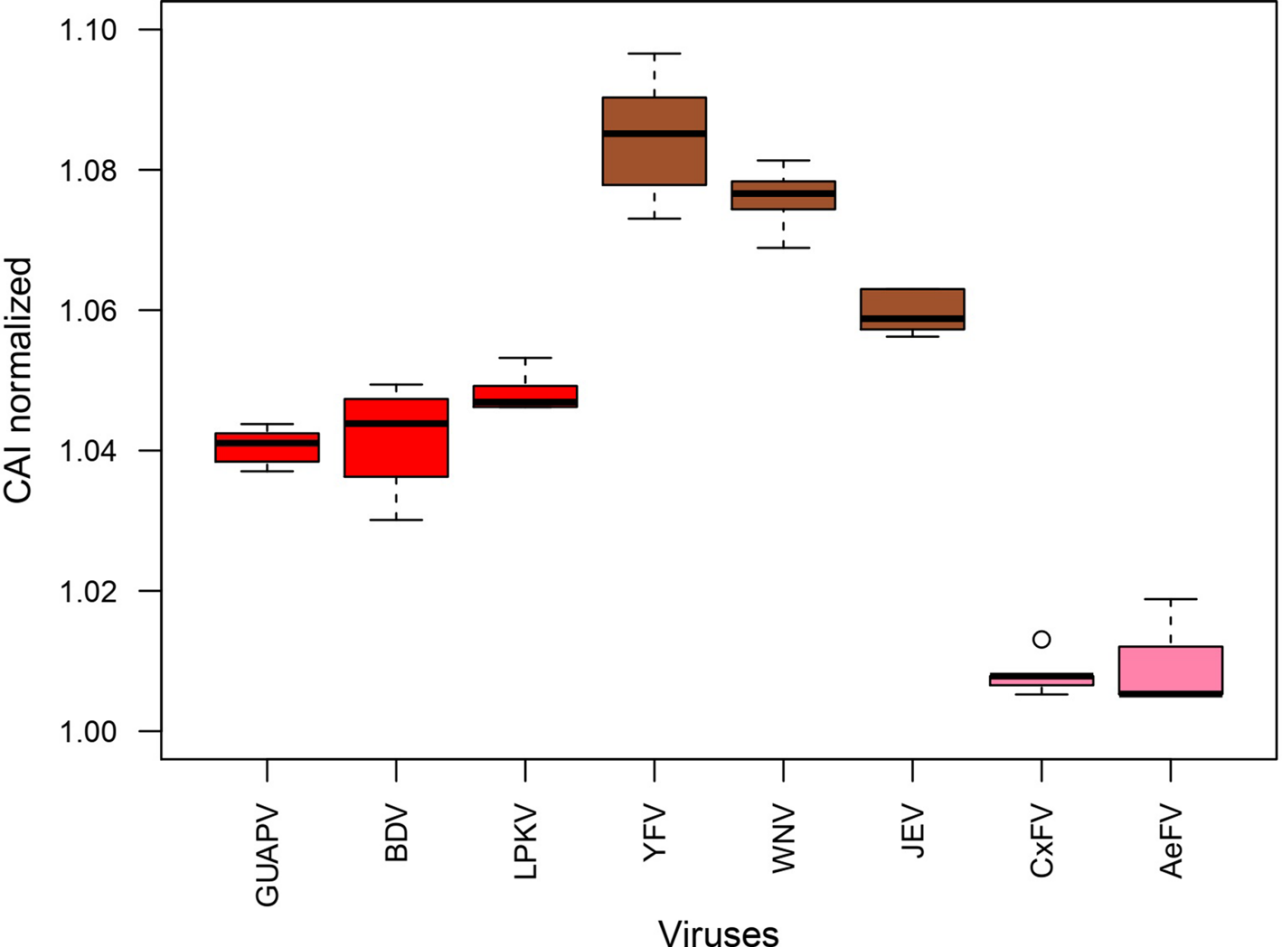
CAI analysis of flavivirus showing signs of codon usage adaptation towards the human host genome. Boxplot for normalized CAI values obtained with polyprotein of dISFV (red box), MBFV (brown box) and cISFV (pink box) was generated using random six strains of each virus. CAI value > 1 was considered as evidence of codon adaptation to human house-keeping genes.

CAI indices were computed in order to compare the codon usage preferences of MBFV, cISFV and dISFV to human house-keeping genes. A normalized CAI (nCAI) ≥ 1.0 indicates that the observed CAI is equal to or greater than the expected value (eCAI); these results could be interpreted as a codon usage adaptation of the *Flavivirus* genus toward human codons. As expected, all cISFV strains analyzed have a nCAI index close to 1, signing a low preference to human genes codons, in contrast, MBFVs strains possess high nCAI values, range from 1.05 to 1.09, confirming their great potential to replicate using human codons (Fig. 5). The nCAI values > 1 were obtained for all GUAPV strains (mean nCAI 1.04), and other dISFV (BDV—mean nCAI 1.04—and LPKV mean nCAI 1.05). Thus, there is evidence that GUAPV, as such other dISFV, could have a higher preference to human codons compared to cISFV. Interestingly, values nCAI observed in dISFV are intermediate in comparison to cISFV and MBFV, and this may represent a potential evolutive preference of dISFV to human codons during the evolutionary history of the *Flavivirus* genus.

### 3′ UTR structures analysis

**Figure 6:**
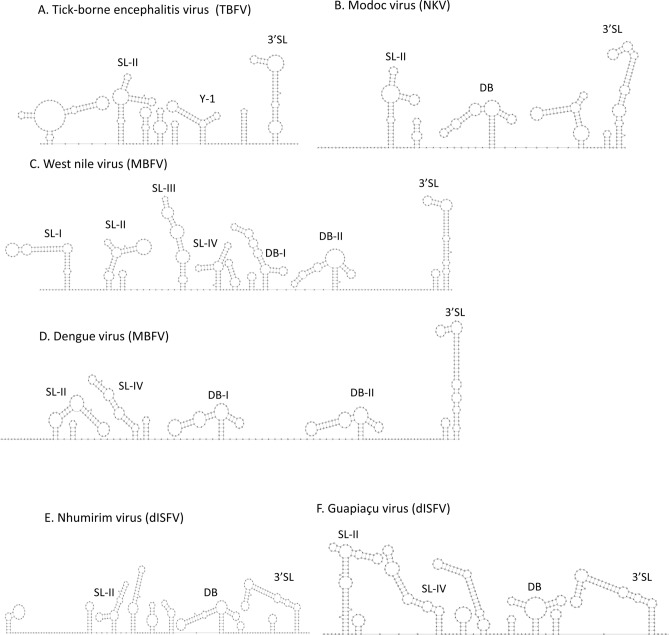
In silico prediction of CFAV, MODV, TBEV, NHUV, WNV and GUAPV 3′UTR and conserved secondary structure. Secondary structures were generated in Mfold software using 3′UTR sequences of each representative strain and structural elements were identified based on previous studies (see “Methods”). SL, stem-loop structure; DB, dumbbell structure; 3′SL, 3′ end stem-loop structure (also named as Flavi_CRE). In silico predicted MBFV xrRNAs-like structures are referred as SL-II and SL-IV, identified by Covariance Models. The structures were visualized in RNAplot software.

Studies of the flavivirus 3′ UTR have identified several secondary structural elements, implicated in multiples viral processes, such as viral replication and translation and inhibition host antiviral response^21^. Prediction in silico modeling of secondary structures based on the energy minimization approach was adopted to identify the structural elements present in GUAPV 3′UTR. Comparison of the GUAPV 3′ UTR and viruses of ISFV, NKV, TBFV, MBFV, and dISFV were made to identify homologous regions of conservation (Fig. 6). GUAPV was identified to have several conserved structural elements in common with viruses in the MBFV group, including a 3′ terminal long-stem loop (3′SL), two conserved stem-loop (SL-II and SL-IV), and a conserved dumbbell-shaped element (DB) (Fig. 6). Secondary structures on 3′UTR flavivirus have shown an action exoribonuclease-resistant (xrRNA) of 5′–3′ Xrn1, an enzyme associated with the cell’s RNA turnover machinery^22^. Partial degradation of viral gRNA by Xrn1 result in the accumulation of long non-coding RNA, called subgenomic flavivirus RNA (sfRNA), and its production may modulate the viral replication, pathogenesis, and cytopathicity^23^.

**Figure 7:**
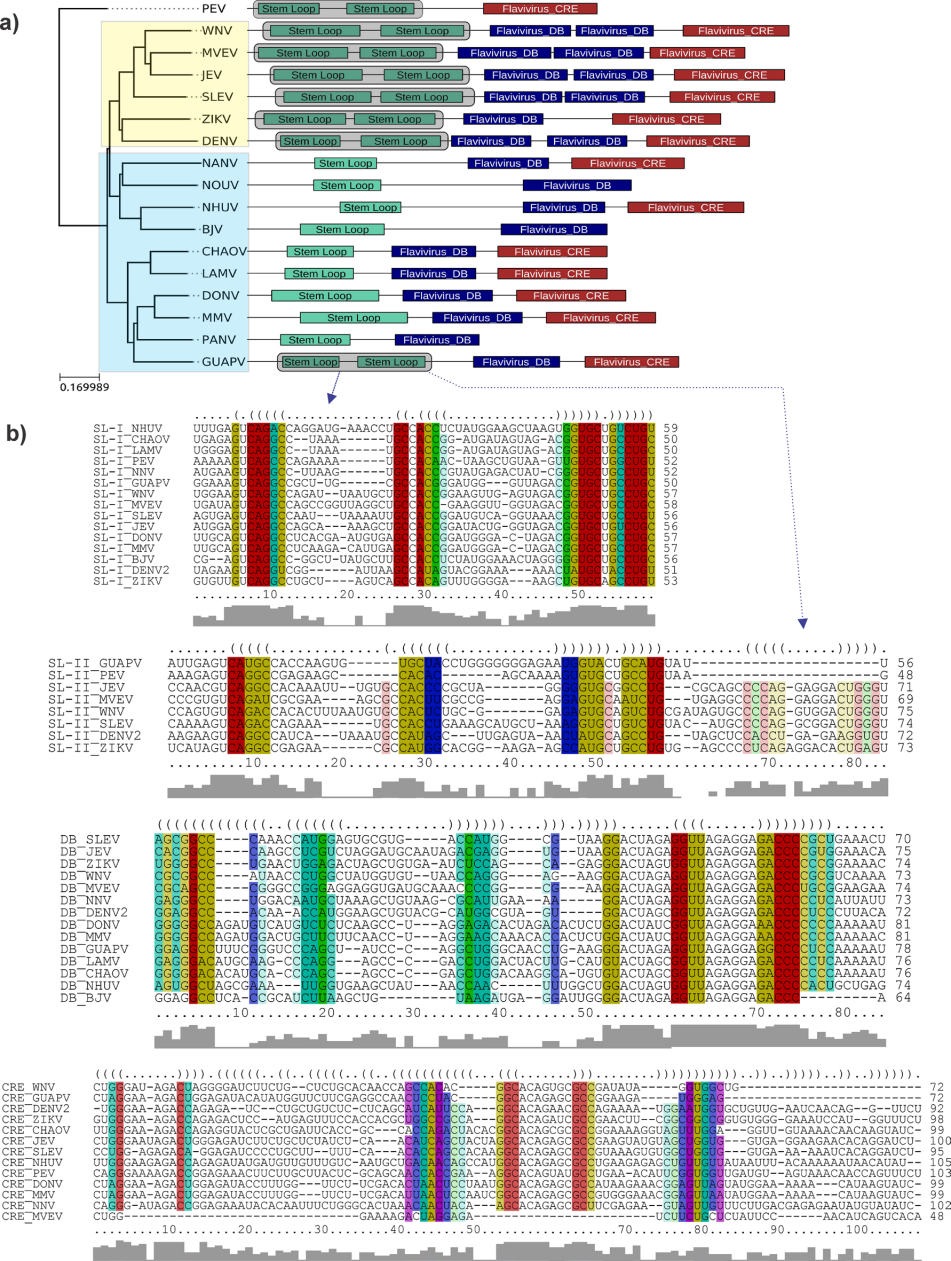
Duplication 3′UTR structures. (**a**) Annotated 3′UTRs of dISFV and MBFV. The phylogenetic tree was constructed using the maximum likelihood method from complete coding sequence nucleotide alignments. MBFV and dISFV strains are highlighted with a yellow box and blue box, respectively. PEV is an outlier. For each species with available 3′UTR sequence, a schematic of the 3′UTR architecture is drawn next to the branches of the tree. Colored boxes represent conserved RNA structural elements identified by CMs, according to implemented in the Infernal package. All boxes correspond to the exact coordinates of annotated structural elements. The gray boxes highlight the duplication of SL in all species in which it occurs. (**b**) Structural alignments of elements of dISFV and MBFV conducted in locRNA^25^.

For a more accurate analysis of RNA secondary structure, a comparative approach to search for homologous RNA structures between MBFV and dISFV was applied using Covariance Model (CM), statistical models of RNA structure that extend classic Hidden-Markov-Models (HMMs) to simultaneously represent sequence and secondary structure^24^. A summary of the RNA elements found in MBFV and dISFV strain is depicted in Fig. 7. The element Flavi_CRE is present in almost all the strains of MBFV and dISFV, including in GUAPV. It is consistently identified as terminal regions within 3′UTRs, and absence of this element from a UTR sequence indicates an incomplete or truncated data, as seen in NOUV, BJV and PANV. Conserved structural blocks of SLs and DBs were identified in all MBFV and dISFV, shown by the green and blue box. In the case of dISFV, single copies of DB and SL elements were observed in most cases, except to GUAPV, in which an additional copy of SL is found (Fig. 7a). The structural alignment of each element found in GUAPV shows structural conservation with MBFV (Fig. 7b). Importantly, the identified host-adaptable SL structure is both conserved and duplicated in all MBFVs, as discussed thereafter.

## Discussion

Here, we report a novel Flavivirus named Guapiaçu virus (GUAPV), isolated from *Aedes* mosquitoes during YFV surveillance studies in Guapiaçu city, SP, Brazil. To our knowledge, this is the first ISFV isolated from *Ae.* *scapularis* and *Ae.* *terrens*. GUAPV sequence was also identified in a human sample from the Amazon Region, in North Brazil.

The diversity of arboviruses in Brazil is vast^26,27^, and some of these viruses are spreading and causing significant epidemics in the country^28,29^. Among these arboviruses, the flavivirus genus includes many pathogenic agents for humans and livestock animals^30^. It was previously proposed that classification within the *Flavivirus* genus should be based on nucleotide similarity of the NS5 gene^3^ and cutoff values were set at 69% for clades and 84% for species. The nucleotide identity, ranging from 59 to 75% in NS5 sequence analysis of ISFVs is used to classify new species^31^. Our strains presented low identity when compared to the closest ISFVs (< 75%), thus suggesting GUAPV should represent a new species. Additionally, GUAPV is grouped in the same clade of the Long Pine Key virus (LPKV) and Marisma mosquito virus (MMV), detected in mosquitoes in Florida, USA, and in Spain, respectively^6,13^ with nucleotide identity in NS5 between 67 to 75%. Interestingly, we detected in this study a GUAPV strain (AP481-2015) in a human plasma sample in Amapá state, north of Brazil. Although GUAPV failed in replicate in mammalian cells, codon usage analysis suggested this virus has a codon bias preference to the human genetic code.

Several new ISFVs have been detected worldwide^14,32–34^, including the Nhumirim virus (NHUV) in Brazil^35^. As for GUAPV, NHUV did not cause a clear CPE in C6/36. However, experiments were conducted with only two cell lines, and the possibility that NHUV might be capable of replicating in other cells is not excluded. Other flaviviruses closely related to NHUV, such as Barkedjii^14^ virus (BJV) also lack a recognized association with vertebrates. Similarly, the observed lack of ability of Lammi virus (LAMV), isolated in Finland, to infect mice and vertebrate cells appear to be contradictory versus its phylogenetic position among the mosquito-borne viruses that are generally associated with vertebrate hosts^18^. A similar contradiction also appears to apply for Nounané virus (NOUV), isolated from *Uranotaenia* mosquitoes in Africa, as it showed little homology to other known flaviviruses once it could be grouped with the pathogenic flaviviruses. However, similar to our findings, NOUV could not replicate in mammalian cells^16^. Differences between observed biological properties and phylogenetic position are also found in the no-known-vector (NKV) flaviviruses Entebbe batvirus (ENTV), Sokuluk virus (SOKV), and Yokose virus (YOKV), since they have no currently known arthropod vectors but group phylogenetically with the mosquito-borne viruses^3,9^. Apparently, these NKV diverged within the MBFV-Aedes spp. associated clade but appear to have lost this mosquito association^36^. A study using reverse genetics has shown that host restriction of Eilat virus (EILV), an alphavirus related to the Western Equine Encephalitis virus complex (WEEV) occurs at levels of entry and replication^37^. More recently, it was described that the ability that an ISFV to infect vertebrates was also blocked during attachment, assembly and release^38^.

Evidence from ongoing metagenomic considers recommends that flavivirus microbes may have developed from before arthropod infections and dISFV can possibly advance the capacity to go about as vertebrate pathogens^11,39^. The number of versatile advances is needed for this to happen is still generally talked about. The most miserly clarification is losing the capacity to contaminate vertebrate cells^37^. In any case, different investigations have been recommended that host range change from single to numerous tropisms probably happened by a few stages in the flavivirus evolutionary history^40^.

Experimental and computational studies have reported that 3′ untranslated region (3′ UTR) may play an important role for host switch in arbovirus. For example, insertion of Sindbis virus (SINV) 3′ UTR motif, an alphavirus that is also an arbovirus, into the 3′ UTR of sleeping disease virus (SDV), an alphavirus that is not able to infect arthropod, increased SDV translation efficiency in insect cell^41^, similarly, insertion of YFV 3′ UTR downstream of NIEV ORF stop codon enhanced translation in BHK cells within the first 6 h post-electroporation^38^. Specifically, 3′ UTR structure duplication has been proposed as an evolutionary feature for MBFV to operate efficiently in organisms phylogenetically distinct with very different antiviral response strategies (mosquito and vertebrate/human)^42^. Our CM analysis shows that stem-loop (SL) and Dumbbell (DB) structure duplication has been present in all MBFV, in contrast to almost all dISFV, which has a single copy of that structure as already reported by Ochsenreiter et al.^43^. The exception is GUAPV, which possesses two copies of SL structure. Evolutionary studies have suggested that tandem RNA structures within DENV 3′UTR are under different selective pressures in mammalian and mosquito hosts, indicating that stem-loop duplication facilities host specialization and result in high viral fitness during host switch^44^. Similarly, evidence for maintaining the primary sequence in duplicated RNA elements has been shown as a possible explanation for ZIKV-induced neurotropism^45^.

As the detection of GUAPV whole genome in a human sample was intriguing, we therefore performed in silico analysis. Codon Adaptation Index (CAI) represents a reliable approach to measure the synonymous codon usage bias and to assess the adaptation of viral genes to their hosts^46^. A difference in translational efficiency for human codon usage preference observed in dual-host (YFV, WNV and JEV) and cISFV (CxFV and AeFV), respectively, could be expected. Interestingly, dISFV (GUAPV, LPKV and BDV) show an intermediated CAI value, indicating a lower effect of the fitness of the virus in a specific host relating codon preferences in comparison to a dual-host flavivirus, however, a recent study analyzing CAI of main groups of flavivirus genus revealed no difference of dISFV strains between insect and vertebrate host preference^47^. This suggests that GUAPV, as such other dISFV, and pathogenic flavivirus are equally or nearly adapted for the human host. Owing to the close relationship to mosquito-borne flaviviruses, dISFV may require fewer adaptive steps to evolve from single to dual tropism than cISFV, as seen in CAI analysis.

In summary, we have described a novel virus phylogenetically more similar to dISFV isolated from two different species of *Aedes* mosquitoes in southeastern Brazil. GUAPV was also detected in a human sample and although this virus did not replicate in mammalian cells, the 3′UTR structure duplication suggests the possibility of host switch, as reported in other arboviruses. This study contributes to a better understanding of the adaptive potential of ISFV to acquire a mechanism to infect different species.

## Methods

### Mosquitoes collection

Due to yellow fever virus (YFV) outbreaks, entomological studies were performed in São Paulo State between October 2016 and March 2017. Mosquitoes were captured by Sucen (Superintendence for Control of Endemic Diseases, State of São Paulo) on ground level by using entomologic net and bottle-type manual vacuums traps. The full description of these mosquitoes pools and sample processing can be found in Cunha et al.^28^. After sampling, they were frozen, transferred to cryogenic tubes, and placed in liquid nitrogen containers for transportation to the laboratory where they were stored in a freezer at − 70 °C until processing. Mosquitoes were identified morphologically, separated into pools according to species and date, and stored at − 70 °C until they were processed.

### Human plasma sampling

Human samples from Amapá State were collected for the viral monitoring program according to the Brazilian Ministry of Health, that is, patients that showed three or more of the following symptoms: high fever that lasts for 2 to 7 days, severe pain in the muscles, bones, and joints, pain behind the eyes, severe headaches, nausea and vomiting, rash, decrease in the number of white blood cells and a low level of platelets in the blood, and/or skin hemorrhages (bleeding under the surface of the skin) that appear as red or purple spots on the body, were tested for Zika, Dengue and Chikungunya virus for diagnosis. Negative samples were tested for pan-flavivirus assay^48^.

All procedures performed in studies involving human participants were in accordance with the ethical standards of the institutional and/or national research committee and with the 1964 Helsinki Declaration and its later amendments or comparable ethical standards. Informed consent was obtained from the adult individual and from all parents or guardians of children participants involved in the study. Ethics Committee approval was granted by Faculdade de Medicina da Universidade de São Paulo (CAAE: 53153916.7.0000.0065), and Centro Universitário Luterano de Palmas—ULBRA (CAAE: 53153916.7.3007.5516). All experiments were performed following safety criteria for virus isolation in Bsl 2 equipment by trained people.

### Viral detection and isolation

Mosquito pools were triturated in sterile grinders containing 1 mL of phosphate-buffered saline solution with 0.75% bovine albumin, penicillin (100 units/mL), and streptomycin (100 µg/mL). The resultant suspension was centrifuged at about 1800*g* for 15 min. The supernatant was withdrawn and frozen at – 70 ºC. Approximately 20 µL of each pool were inoculated into cell tubes containing monolayer cultures of C6/36 cells (CEIAL 062) with 10% FBS. After medium removal and adsorption for 1 h, tubes were incubated for 9 days at 28 °C with L-15 medium with 2% FBS, penicillin (100 units/mL) and streptomycin (100 µg/mL) at 28 ºC. Indirect immunofluorescent assays (IFA) were performed using a pool of in-house anti-dengue virus (DENV1-4) hyperimmune polyclonal antibodies a FITC-labeled anti-mouse IgG (whole molecule) antibody (Sigma-Aldrich, Missouri), and later with anti-DENV and anti-YFV monoclonal antibodies provided by Centers for Disease Control and Prevention (CDC) if positive for *Flavivirus*^49^*.* Supernatants from positive IFA samples that were negative for DENV and YFV using monoclonal antibodies were extracted using QIAamp RNA Viral Mini Kit (QIAGEN, Hilden, Germany), according to manufacturer’s instructions followed by RT-qPCR for ZIKV, WNV, and for *Flaviviru*s genus^48,50^.

Viral RNA from the plasma samples was extracted using a MagNa Pure 2.0 Roche automatic nucleic acid extraction machine (MagNA Pure LC instrument, Roche Applied Science, Indianapolis, Ind.). The reagent kits used for extraction were from the MagNa Pure LC Total Nucleic Acid Isolation Kit- High Performance, Version 8, by Roche (Roche Applied Science, Indianapolis, Ind.); the protocol used was that specified by the kit instructions. 200 μL of blood plasma was used from each sample for extraction; if the sample did not have a full 200 μL of volume, PBS was added to the sample up to a total volume of 200 μL and the contents of the sample flasks were agitated gently with a pipette. The final elution volume for each sample was 60 μL. After extraction, the samples were stored in a – 80 ºC degree freezer. The sample was then submitted to a series of qPCR assays; first, the ZDC (Zika, Dengue, Chikungunya) Multiplex qPCR Assay, by BIORAD (Bio-Rad Laboratories, Inc.; Hercules, California) was applied. The assay was performed according to the manufacturer’s protocol, which is specified in the kit; 5 μL of extracted RNA was used for the assay. The samples that showed negative results for the ZDC assay were then submitted to a pan-Flavivirus multiplex qPCR assay, using the primers and protocol described by Patel et al.^48^. Again, 5 μL from each sample of extracted RNA was used for the assay. The samples negative for ZDC and positive for pan-flavivirus RT-qPCR were submitted for metagenomic NGS protocol.

### IFA experiments and viral growth in cell culture

First, in order to check cross-reaction within different hyperimmune polyclonal antibodies, a second passage (C6/36) of GUAPV was inoculated into C6/36 cells as described above, and IFA tests were performed using an in house anti-YF BeH-111 strain, anti-SLEV Span-11916 strain and anti-DENV3 hyperimmune polyclonal antibodies. All antibodies showed positive labeling with similar immunofluorescence signals under the immunofluorescence microscope, although anti-SLEV showed the strongest signal (data not shown).

Later, 20 µl of GUAV second passage (C6/36) were inoculated into 12-well cell culture plates containing Hela cells (CEIAL 001), RD cells (CEIAL 039), Vero cells (CEIAL 057) and C6/36 cells (CEIAL 062) after medium removal. C6/36 plates were incubated at 28 °C with L-15 medium with 2% FBS, penicillin (100 units/mL) and streptomycin (100 µg/mL) at 28 °C, while Hela, RD and Vero cells were incubated at 37 °C, 5% CO_2_, using mediums Eagle, Eagle + L15 and 199, respectively, with 2% FBS, penicillin (100 units/mL) and streptomycin (100 µg/mL). Plates were checked daily for cytopathic effect, and supernatants of each cell were harvested every 24 h during 7 days and stored at – 80 ºC until use. In the 7th day, cells were harvested using cell scraper, and an IFA tests were performed^49^. All supernatants were extracted using QIAamp RNA Viral Mini Kit (QIAGEN, Hilden, Germany) according to the manufacturer’s instructions, followed by a pan-flavivirus RT-qPCR^48^.

### Next-generation sequencing

Whole genomes were obtained from C6/36 cell infected and human plasma positive for pan-flavivirus RT-qPCR. The protocol used to perform deep sequencing was described previously by da Costa et al.^29^. Briefly, 500 μl of each sample was homogenized in a 2 ml impact-resistant tube containing lysing matrix C (MP Biomedicals, CA). The homogenized sample was centrifuged at 12,000×*g* for 10 min, and approximately 300 μl of the supernatant was then filtrated through a 0.45 μm filter (Merck Millipore, Billerica, MA). Approximately, 100 μl of cold PEG-it Virus Precipitation Solution (System Biosciences, CA, USA) was added to the obtained filtrate, mixed and incubated at 4 °C for 24 h. After the incubation period, the mixture was centrifuged at 10,000×*g* for 30 min at 4 °C and the supernatant discarded. The pellet rich in viral particles was treated with a mix of nuclease enzymes (TURBO DNase and RNase Cocktail Enzyme Mix-Thermo Fischer Scientific, CA, USA; Baseline-ZERO DNase—Epicentre, WI, USA; Benzonase-Darmstadt, Germany; and RQ1 RNaseFree DNase and RNase A Solution-Promega, WI, USA) to digest unprotected nucleic acids. Viral nucleic acids were then obtained using ZR & ZR-96 Viral DNA/RNA Kit (Zymo Research, CA) according to the manufacturer’s protocol and cDNA synthesis was performed using SuperScript III (Thermo Fischer Scientific, CA) and random decamer (Thermo Fischer Scientific, CA). The second strand of cDNA synthesis was obtained using DNA Polymerase I Large Fragment (Promega, WI). Then, cDNA library analysis was performed using Nextera XT Sample Preparation Kit (Illumina, CA, USA). The library was deep-sequenced using the HiSeq 2500 Sequencer (Illumina, CA) with 126 bp ends. RNA reads have been deposited at NCBI, Bioproject accession number PRJNA678702.

### Contigs assembly

Bioinformatic analysis was performed according to the protocol previously described by Deng et al.^51,52^. Briefly, the non-viral sequences (i.e. human, bacterial, and fungal sequences) were removed using bowtie2. Later, the unmapped sequences were used for the reconstruction of viral genomes using an ensemble assembler, including SOAPdenovo2 (available at ftp://public.genomics.org.cn/BGI/SOAPdenovo2), Abyss (available at http://www.bcgsc.ca/platform/bioinfo/software/abyss.), meta-Velvet (available at http://metavelvet.dna.bio.keio.ac.jp/), CAP3 (available at http://www.mrc-lmb.cam.ac.uk/pubseq), Mira (https://sourceforge.net/projects/mira-assembler/files/MIRA/) and SPADES (http://cab.spbu.ru/software/spades/) programs. The resulting singlets and contigs were analyzed using BLASTx to search for similarity to viral proteins in GenBank’s Virus RefSeq. Also, the contigs were compared to the GenBank nonredundant nucleotide and protein database (BLASTn and BLASTx). The Sequences obtained with de novo assembly and identified in blast were then submitted mapping with the Geneious R9 Software (Biomatters Ltd L2, 18 Shortland Street Auckland, 1010, New Zealand), so that the generated sequence did not generate a biased or chimera sequence.

### Genomic properties analysis

The nearly complete genomes of GUAPV was analyzed to determine the open read frame (ORF) and peptides region by potential cleavage sites identified using the SignalP 3.0 software (http://www.cbs.dtu.dk/services/SignalP/). The nucleotide and amino acid identity among representants of flavivirus were calculated for the complete ORF and all peptides of coding sequence (CDS) by the identity matrix tool of BioEdit 7.0.5.3 software^53^. The potential N-glycosylation sites were predicted for GUAPV proteins using the NetNGlyc 1.0 server (available at http://www.cbs.dtu.dk/services/NetNGlyc/).

### Phylogenetic analysis

The entire ORF was aligned with other flavivirus sequences available at the public database (NCBI) using the Mafft software program (version 7.0.9). The General Time-reversible (GTR) model substitution was choose in jModelTest software^54^ and phylogenetic tree was estimated using a maximum-likelihood analysis using the PhyML software^55^. Support for nodes was assessed using approximate Likelihood-Ratio Test (aLRT) and tree was visualized in FigTree software. Additionally, the genetic distance between and within dISFV I and dISFV II clades were compute using p-distance implemented in MEGA X software^56^.

### Codon Adaptation Index to human house-keeping genes

The Codon Adaptation Index (CAI) is a measure of the synonymous codon usage bias making comparisons of codon usage preferences in different organisms and assessing the adaptation of viral genes for a given hosts^57^. Thus, the CAI of flavivirus for housekeeping of human genes was calculated, according by Nicoholas Di Paola^47^. First, we obtain the “raw” CAI value (rCAI) using CAIcal program^46^, and the expected-CAI (eCAI) value based on 1000 random viral sequences with similar length, codon composition, GC-content and human amino acid usage was calculated using e-CAIcal program^58^, then a normalized CAI (nCAI) threshold was obtained by calculating rCAI/eCAI values. A value above ‘1’ is considered as evidence of codon adaptation to the reference set of codon preferences.

### RNA structure prediction and structural homology search of GUAPV 3′UTR

The 3′ UTR of the GUAPV were compared to 3′ UTRs of representative members from other flaviviruses representing the distinctive phylogenetic and phenotypic grouping viruses in order to identify homologous secondary structures and repeat elements that could associate with phylogenetic or phenotypic patterns. The structural elements and sequences of secondary structure RNA were identified in direct comparison from previous studies^59–62^. A consensus sequence of 3′UTR was used for secondary structure prediction of GUAPV in Mfold webserver (http://unafold.rna.albany.edu/?q=mfold/RNA-Folding-Form). Two MFold parameters, i.e. maximal distance between paired bases of 80 and flat exterior loop type, were calibrated manually, as previously described by Gritsun et al.^63,64^. The secondary RNA structure was visualized using the RNAplot^65,66^.

To localize RNA homologous structures in flavivirus 3′UTRs, we used Covariance Models (CM) implemented in Infernal package for conserved RNA elements^24^. CMs allow for rapid screening of large RNA sequence databases to conserved sequence-only or structurally homologous RNAs. To this end, we obtained the CM of RNA structure families of flavivirus from Rfam database: Flavivirus_DB (Rfam ID: RF00525), Flavivirus 3′ UTR cis-acting replication element (Flavi_CRE) (Rfam ID:RF00185), flavivirus capsid hairpin cHP (Rfam ID: RF00617), Flavivirus 3′UTR stem loop (SL) (Rfam ID: RF01415), Japanese encephalitis virus hairpin structure (Rfam ID: RF00465) and Pseudoknot PSK3 (Rfam ID: RF02549). Then, homologies of both sequences and secondary structures were inferred by CMs as implemented in the Infernal package. Additionally, a structural alignment of the homologous structures found in GUAPV was conducted in LOCARNA software^67^.

## Supplementary Information

Supplementary Information.

## Data Availability

The datasets generated during and/or analysed during the current study are available from the corresponding author on reasonable request.
