# Supplementary material for: Guapiaçu virus, a new insect-specific flavivirus isolated from two species of Aedes mosquitoes from Brazil
Source: Sci Rep. 2021 Feb 25;11:4674. doi: 10.1038/s41598-021-83879-6 (PMC7907106; doi:10.1038/s41598-021-83879-6)

Suplementary material

S1: Guapiaçu vírus growth kinetics in Vero, RD, Hela and C6/36 cell lines (Days p.i in X-axis, Ct values in Y-axis).

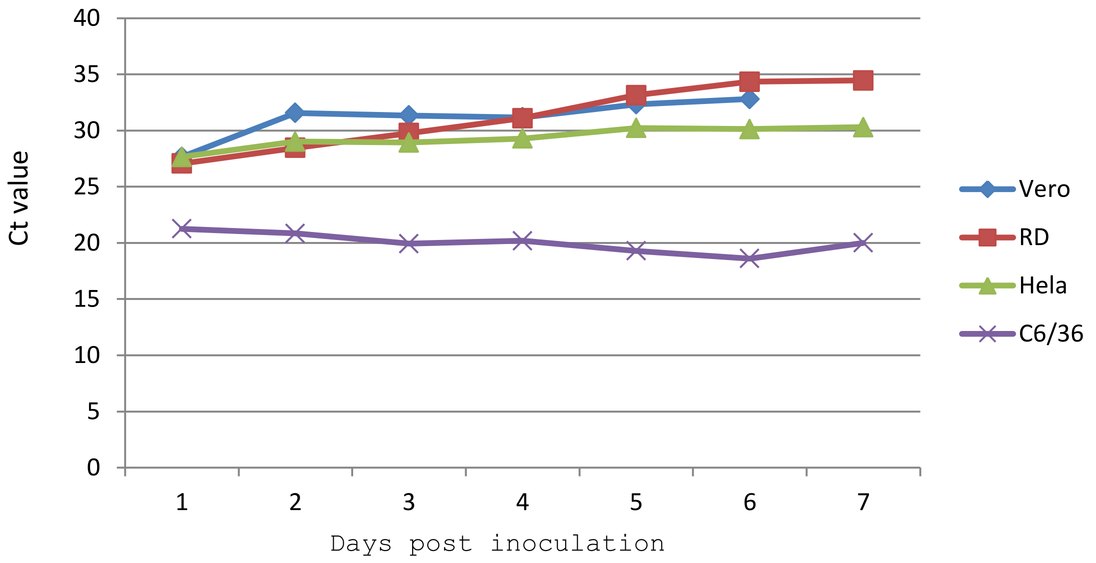

S2: Boxplot of Ct values distribution of Guapiaçu virus in Vero, RD, Hela and C6/36 cells. The thick horizontal line indicates the median. Grey boxes and vertical lines indicate interquartile range and the variance between Ct values per cell type, respectively.

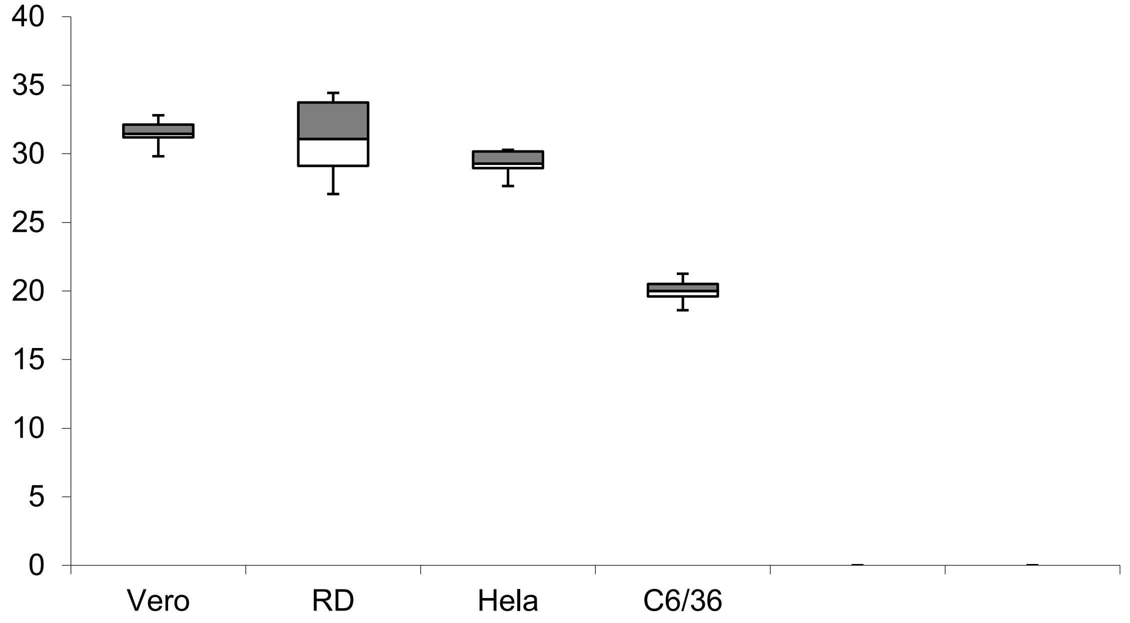

Supplement: Supplementary file 1 — Supplementary Information. [file 41598_2021_83879_MOESM1_ESM.pdf]
